# Cognitive Performance and Mood Following Ingestion of a Theacrine-Containing Dietary Supplement, Caffeine, or Placebo by Young Men and Women

**DOI:** 10.3390/nu7115484

**Published:** 2015-11-19

**Authors:** Daniel J. Kuhman, Keanan J. Joyner, Richard J. Bloomer

**Affiliations:** Cardiorespiratory/Metabolic Laboratory, School of Health Studies, University of Memphis, Memphis, TN 38152, USA; kuhmand15@students.ecu.edu (D.J.K.); joyner@psy.fsu.edu (K.J.J.)

**Keywords:** dietary supplements, theacrine, reaction time, caffeine, trail making test, digit symbol substitution test

## Abstract

Theacrine is a purine alkaloid found primarily in the leaves of the Camellia Kucha plant and is now included within dietary supplements. To compare the effects of a theacrine-containing dietary supplement with caffeine and placebo on energy and mood, as well as objective measures of cognitive performance, heart rate, and blood pressure, 10 healthy men (20.8 ± 0.7 years) and 10 healthy women (22.2 ± 1.1 years) ingested the dietary supplement TheaTrim (Purus Labs; containing a branded form of theacrine (Teacrine™) and caffeine (150 mg)), caffeine only (150 mg), or a placebo on three different days, separated by approximately one week. Before, and for up to 4 h following, ingestion of the assigned condition, subjects completed a subjective assessment of energy and mood, as well as tests of cognitive performance (trail making test (TMT), digit symbol substitution test (DSST)), and reaction time. Heart rate and blood pressure were measured. No condition or interaction effects were noted for TMT, DSST, or reaction time, despite a trend for improvement in selected variables with both TheaTrim and caffeine treatment. Condition effects or trends were noted for subjective feelings, with values for attentive, alert, focused, and energetic higher for TheaTrim than for placebo and caffeine, while values for lethargic and groggy were lower for TheaTrim than for placebo and caffeine. Heart rate and blood pressure were largely unaffected by treatment. These data indicate that TheaTrim treatment does not result in a statistically significant improvement in cognitive performance but may favorably impact multiple subjective feelings related to energy and mood.

## 1. Introduction

The use of dietary supplements to increase energy and focus is commonplace, as evidenced by the widespread sale and consumption of energy drinks and energy shots, as well as capsule-based dietary supplements. This use is particularly popular among consumers in the 18–35 years old category and may be associated with relatively unhealthy dietary intake [1]. Recently, a novel plant-based ingredient has made its way into the dietary supplement market: a purine alkaloid found primarily in the leaves of the Camellia Kucha plant known as theacrine [2].

Theacrine (1,3,7,9-tetramethyluric acid) is thought to act through both the adenosine and dopamine systems to provide a mild stimulant effect, as well as a calming effect. It is structurally similar to caffeine and has been reported to have antioxidant [3], anti-inflammatory/analgesic [4], anti-depressive [5], locomotor [6], and sedative/hypnotic properties [7]. Collectively, this agent may have wide-ranging effects in human subjects but to date has only been evaluated in two pilot studies [8,9]. The initial two-part pilot study involving 15 human subjects found that theacrine (Teacrine™, Compound Solutions, Inc., Carlsbad, CA, USA) delivered at an acute dose of 200 mg promoted a significant increase in energy, a reduction in fatigue, and a trend (*p* = 0.07) towards improved concentration (based on a subjective questionnaire) compared to a placebo group. The same study included data for a group of six subjects who were dosed 100, 200, or 400 mg of theacrine over a seven-day period where they noted moderate to large effect sizes for energy, fatigue, concentration, anxiety, motivation to exercise, and libido with the 200 mg dose [8]. However, in the latter portion of this study, it is difficult to draw firm conclusions about the differential dosage effects using such a small sample of subjects and only seven days of treatment. In a more recent study, 60 young and healthy men and women received either a placebo or theacrine (Teacrine™) at a dosage of 200 or 300 mg per day for a period of eight weeks to determine the safety profile of this agent. No changes of statistical significance were noted for any safety measure [9]. Although these initial studies related to potential efficacy and safety are promising, more research using this agent is needed. 

While the impact of theacrine on perceived energy and mood remains to be firmly determined, one agent with fairly strong evidence to support its efficacy is caffeine. Chemically known as 1,3,7-trimethylxanthine, caffeine is known to improve attention [10] and may improve cognitive performance and subjective feelings of energy and mood [11]. Smit and Rogers showed that caffeine significantly sped up reaction time, improved performance in habitually high caffeine consumers during a rapid visual information processing (RVIP) task—a task that places a high load on working memory, and increased the subjective mood factor “energetic arousal” [12]. Herz also found that subjects receiving caffeine reported higher levels of arousal compared to those receiving a placebo [13]. More recently, functional magnetic resonance imaging (fMRI) data have shown that caffeine may increase the working memory process both directly, as well as indirectly, via arousal modulation [14]. It is possible that the combination of theacrine and caffeine may have more profound effects on aspects of cognitive performance and subjective feelings of energy and mood as compared to either when consumed alone. 

Considering the above, the present study was designed to compare a theacrine-containing multi-ingredient dietary supplement (containing caffeine) with a caffeine only condition, as well as with a placebo condition on measures of cognitive performance, reactive time, subjective feelings of energy and mood, and heart rate and blood pressure in young men and women. 

## 2. Experimental Section

### 2.1. Subjects

Twenty young and healthy men (*n* = 10) and women (*n* = 10) between the ages of 18 and 31 years participated in this study. We chose to retain a relatively young group of homogenous subjects knowing that this group is most likely to consume dietary supplements designed to enhance energy, in addition to the fact that cognitive ability often decreases with age [15]. No woman was pregnant or nursing and all women were required to perform a urine pregnancy test on each day of testing to confirm that they were not pregnant. It should be noted that no power calculations were performed in the present study due to the scarcity of prior data and effect sizes for the subjective measures, as well as the exploratory nature of this work and the inclusion of various outcome measures.

All subjects were regular consumers of caffeine (*i.e.*, caffeine intake two or more days per week), through beverages (e.g., coffee, tea, and energy drinks) or dietary supplements. This minimum consumption of caffeine was chosen to ensure that participants were familiar with how caffeine affected them and that they would not react adversely to the consumption of caffeine in the non-placebo conditions. Subjects could not be hypertensive (resting blood pressure ≥ 140/90 mm Hg). Subjects were not current smokers and did not have a history of substance abuse. In addition, subjects did not have diagnosed cardiovascular, metabolic, neurological, or mental disorders such as heart disease, diabetes, seizures, anxiety, depression, attention deficit disorder (ADD), and/or attention deficit hyperactivity disorder (ADHD). Finally, subjects could not have been using any medication that might impact testing. Health history, medication and dietary supplement usage, and physical activity questionnaires were completed by all subjects. Prior to participation, each subject was informed of all procedures, in accordance with the procedures approved by the University of Memphis Institutional Review Board for Human Subjects Research (protocol #3566). Subjects provided written informed consent prior to participating. 

### 2.2. Initial Laboratory Visit: Screening Visit

During the initial visit to the laboratory, subjects completed the informed consent form, health history, and physical activity questionnaires. Subjects’ heart rate (radial pulse) and blood pressure (via auscultation), height, weight, waist, and hip circumference were measured. Subjects completed a familiarization trial for each of the cognitive performance tests described below, as well as for the reaction time test. Following completion of the screening, subjects were scheduled for their initial testing visit. 

### 2.3. Assignment of Conditions

Subjects were randomly assigned in double-blind manner using a cross-over design to one of three conditions. Subjects reported to the lab on three different days (separated by approximately 1 week) and received one of the below conditions on each day. The conditions were consumed within the presence of an investigator, with 12 ounces of water. (1) TheaTrim (Purus Labs, Dallas, TX, USA); (2) caffeine; or (3) placebo. The TheaTrim contained a branded form of theacrine (Teacrine™; Compound Solutions, Inc., Carlsbad, CA, USA), caffeine (150 mg), white willow bark, SAMe, Rauwolfia vomitoria extract, citrus bioflavonoid complex, and vitamin B12. The caffeine condition contained 150 mg of caffeine, to match the content in the TheaTrim. Due to the proprietary nature of the TheaTrim product, the exact amount of theacrine cannot be disclosed but was similar to the amount that has been used in prior human studies involving theacrine. The placebo contained cellulose powder. All capsules were provided to investigators in unlabeled bottles and the blinding code was held by the contract manufacturer until all data collection was completed. 

### 2.4. Test Visit Procedures

Subjects reported to the lab a total of three times during the morning hours. Testing began at approximately 6:00–8:00 a.m. for each visit. The early time of day was chosen so that we could likely ensure that subjects were not reporting to the lab in a fed state and were not yet presented with daily challenges that might have influenced outcomes (*i.e.*, exposed to significant mental or physical stress). We have maintained a similar data collection time in many other studies with success. While the time was early for some participants, the time of day was matched for all three visits, limiting the possibility of variance in response from day to day. Subjects reported to the lab following an overnight fast (at least 8 h), without having consumed alcohol or caffeine within the past 24 h. Subjects were also instructed to obtain at least 7 h of sleep during the night prior to testing.

Upon arrival at each visit, subjects were asked to void and then rested quietly for 20 min and then had baseline (pre meal) data collected, as described below for testing battery. Following this they received a standardized meal of approximately 200 calories (8 ounces orange juice and 20 g of vanilla protein powder; Purus Labs MyoFeed). The standardized meal was administered to ensure the comfort and safety of our subjects and, combined with the prior fasting, allowed us to control for possible digestion confounds (*i.e.*, everyone consumed the pills under the same internal conditions). Subjects then rested quietly for 30 min and post meal data were collected. Subjects were then provided with their assigned condition (as described above) and data were again collected at 1, 2, 3, and 4 h post ingestion. Therefore, all subjects completed the testing battery a total of six times during each lab visit. No other food or calorie-containing beverages were provided during the study period. Subjects were allowed to consume as much water as desired and the amount was matched for each visit. Subjects relaxed in the lab and watched TV, used the computer, listened to music, or read during the assessment period. 

### 2.5. Testing Battery

The following data were collected from subjects at baseline (pre meal), post meal (before condition intake), and at 1, 2, 3, and 4 h following condition intake. During the immediate 5 min before beginning testing at each of the testing times, subjects were seated quietly in a chair. Collected data included: Heart rate, Blood pressure, Reaction time test, Appetite assessment, Subjective feelings questionnaire (ranging from 1 (none) to 10 (extreme)), Digit Symbol Substitution Test (DSST), and Trail Making Test (TMT).

Subjects’ reaction time was assessed using a computerized test program. The program displayed a different color light queue and subjects responded to the queue as quickly as possible by pressing a keyboard key. Five trials were performed at each measurement time and the mean of the five trials was used in data analysis. The subjective feelings questionnaire included commonly used marketing terms specific to energy supplements. Rather than use an existing tool that may not have been specific to the outcome variables of interest, we chose to create a questionnaire that would be highly specific to the items of interest related to use of the tested products/ingredients. The TMT [16] and DSST [17] are commonly used as assessments of cognitive performance and provide numeric scores, which can then be used as objective indicators of condition effectiveness. Two practice trials were administered to subjects for both the DSST and the TMT, and some learning effects are expected [18,19]. Different versions of each test were provided to subjects at all assessment times and attempts were made to provide equal difficulty for all tests. Variations of the Trail B test were made to include a stable measure of cognitive flexibility that could be administered 18 times. Pilot testing was performed using the variants of the tests to ensure that tests were of similar difficultly. 

For the DSST, subjects were provided with two minutes to record as many correct matching digits and symbols as they could. Specifically, they were instructed to record the matching digit in the box under the correct corresponding symbol. A key was provided to note the matching digits and symbols. Subjects were required to complete one entire row before moving on to the next row. Errors in matching were not counted against the subject but only correct scores were counted in the total for each trial. Salthouse and colleagues demonstrated ample construct validity of the DSST, which has emerged as a routine tool for assessing cognitive ability and working memory [20]. 

For the TMT, subjects completed only part B of the traditional test. This included connecting numbers and letters from beginning to end (from low to high; 1–13 and A–L), as quickly as possible. Subjects were provided with detailed instructions to alternate their tracing pattern between a number and letter in the proper order. If a mistake was made, investigators stopped the participant, instructed them to go back to the circle where the mistake was made, and continued the test. Time to completion represents the total time for each testing trial, including errors. The Trail B test also exhibits exceptional construct validity. Extant research has shown impairment on the Trail B test due to concussion [21], chronic schizophrenia [22], and aging [23]. These data demonstrate the expected results from physical impairment on a cognitive flexibility measure, and are in agreement with data on cognitive decline due to aging, showing the Trail B test to be an appropriate measure to use in the current study. That is, younger and healthy individuals should be highly capable of performing well on this test. Our use of a relatively homogenous sample of subjects, all young and otherwise healthy, reduced significantly the potential for large subject–subject variance in this measure. 

### 2.6. Dietary Records and Physical Activity

All subjects were instructed to maintain their normal diet during the study period and were asked to record all food and drink consumed during the day prior to each test day. Subjects were asked to match food intake for each day of recording, so that dietary intake was not different on the days prior to each test day. Records were analyzed for total kilocalorie, macro- and micro-nutrient composition using Food Processor (version 9.9; ESHA Research, Salem, OR, USA).

### 2.7. Statistical Analysis

All data were analyzed using a 2 (sex) × 3 (condition) × 4 (time) repeated measures analysis of variance (ANOVA) in an attempt to highlight any sex-related differences in treatment. Significant effects were analyzed further with *post hoc* testing, using the conservative method of Tukey due to the smaller sample size. A one-way ANOVA was used to analyze dietary intake during the day prior to each condition assignment. All analyses were performed using JMP statistical software (version 4.0.3, SAS Institute, Cary, NC, USA). Statistical significance was set at *p* ≤ 0.05.

## 3. Results

### 3.1. Baseline Characteristics, Dietary Intake, and Appetite

All 20 subjects successfully completed the study; however, data were not obtained for one male subject during the placebo condition at 2 h collection time (due to an oversight on the part of the investigators). Subject characteristics are presented in Table 1. As expected, some differences were noted between men and women for the various characteristics assessed (e.g., height, weight, and waist circumference). Dietary data are presented in Table 2 and differences were noted between men and women for each variable, as follows: kilocalories (*p* < 0.0001), protein (*p* < 0.0001), carbohydrate (*p* = 0.04), fat (*p* = 0.001), vitamin C (*p* = 0.006), vitamin E (*p* < 0.0001), vitamin A (*p* < 0.0001), and water intake (*p* = 0.04). No other differences of statistical significance were noted for any variable (*p* > 0.05).

**Table 1:** Characteristics of 20 healthy men and women ingesting TheaTrim, placebo, or caffeine.

Variable	Men (*n* = 10)	Women (*n* = 10)	*p* Value
Age (years)	20.8 ± 0.7	22.2 ± 1.1	0.28
Height (cm)	180.9 ± 1.7	167.7 ± 2.5	0.00
Body Weight (kg)	86.8 ± 5.5	74.9 ± 2.9	0.01
Body Mass Index (kg·m^−2^)	26.4 ± 1.2	23.9 ± 0.8	0.10
Waist Circumference (cm)	86.4 ± 3.0	74.9 ± 1.6	0.00
Hip Circumference (cm)	103.8 ± 2.3	99.0 ± 2.1	0.15
Waist:hip	0.83 ± 0.01	0.76 ± 0.01	0.00
Resting Heart Rate (bpm)	68.5 ± 3.1	71.8 ± 4.5	0.55
Resting Systolic Blood Pressure (mmHg)	129.1 ± 1.9	118.2 ± 3.8	0.02
Resting Diastolic Blood Pressure (mmHg)	69.8 ± 2.9	70.6 ± 2.1	0.83
Average Daily Caffeine Intake (mg)	125.9 ± 20.6	123.5 ± 39.6	0.96
Weekly Aerobic Training (h)	1.7 ± 0.7	2.7 ± 0.6	0.31
Aerobic Training History (years)	4.4 ± 1.5	5.0 ± 1.6	0.77
Weekly Anaerobic Training (h)	5.9 ± 0.8	1.4 ± 0.4	0.00
Anaerobic Training History (years)	4.2 ± 0.8	2.4 ± 1.0	0.20

Values are mean ± SEM (standard error of the mean).

**Table 2:** Dietary data of 20 healthy men and women during the 24 h prior to ingesting TheaTrim, placebo, or caffeine.

Variable	TheaTrim	Placebo	Caffeine
Kilocalories *	2667.7 ± 160.7	2713.2 ± 346.1	2723.6 ± 309.0
*men*			
Kilocalories	1804.3 ± 120.8	1694.6 ± 157.2	1869.2 ± 136.0
*women*			
Protein (g) *	165.9 ± 20.2	166.5 ± 22.6	168.2 ± 24.0
*men*			
Protein (g)	79.0 ± 11.3	78.6 ± 11.6	83.5 ± 12.2
*women*			
Carbohydrate (g) *	249.3 ± 26.4	271.1 ± 65.0	262.8 ± 35.4
*men*			
Carbohydrate (g)	211.4 ± 11.6	187.5 ± 22.7	205.1 ± 21.6
*women*			
Fat (g) *	112.5 ± 11.0	106.6 ± 13.8	111.4 ± 22.3
*men*			
Fat (g)	72.0 ± 8.2	71.6 ± 7.0	81.7 ± 7.1
*women*			
Vitamin C (mg) *	123.5 ± 35.1	151.5 ± 42.9	115.4 ± 23.8
*men*			
Vitamin C (mg)	70.5 ± 20.7	43.5 ± 10.6	77.1 ± 24.4
*women*			
Vitamin E (mg) *	12.5 ± 3.1	10.0 ± 2.8	12.5 ± 3.2
*men*			
Vitamin E (mg)	2.3 ± 0.7	1.2 ± 0.4	2.1 ± 0.4
*women*			
Vitamin A (RE) *	1092.3 ± 284.4	1140.4 ± 310.4	1260.9 ± 333.2
*men*			
Vitamin A (RE)	390.1 ± 152.4	222.6 ± 90.8	377.0 ± 142.3
*women*			
Water (in lab; ounces) *	23.9 ± 3.8	24.3 ± 3.7	24.1 ± 3.8
*men*			
Water (in lab; ounces)	18.0 ± 4.0	18.0 ± 4.0	16.8 ± 2.8
*women*			

Data are presented separately for men and women to best inform readers about how each sex responded. Values are mean ± SEM (standard error of the mean). * Sex effect noted for kilocalories (*p* < 0.0001), protein (*p* < 0.0001), carbohydrate (*p* = 0.04), fat (*p* = 0.001), vitamin C (*p* = 0.006), vitamin E (*p* < 0.0001), vitamin A (*p* < 0.0001), and water intake (*p* = 0.04); No other differences of statistical significance were noted (*p* > 0.05). RE: retinol equivalents.

Regarding appetite, a sex effect (*p* < 0.0001; higher in men compared to women) and time effect was noted (*p* < 0.0001; appetite at Hours 3 (6.3) and 4 (7.5) higher than at Hours 1 (4.2) and 2 (5.2), as well as pre meal (4.7) and post meal (3.5)).

### 3.2. Subjective Feelings

Regarding subjective feelings, a condition effect was noted for groggy (*p* = 0.04), with values lower for TheaTrim than for placebo and caffeine. A trend was noted for a condition effect for lethargic (*p* = 0.09), with lower values for TheaTrim than for placebo and caffeine. A condition effect was noted for jittery (*p* = 0.01) and depressed (*p* = 0.02), with values higher for caffeine than for TheaTrim and placebo. A trend was noted for a condition effect for attentive (*p* = 0.06), alert (*p* = 0.06), focused (*p* = 0.11), and energetic (*p* = 0.06), with values for TheaTrim higher than for placebo and caffeine.

Sex effects were noted for the following variables: attentive (*p* = 0.0003; higher for men), alert (*p* = 0.0005; higher for men), focused (*p* < 0.0001; higher for men), energetic (*p* < 0.0001; higher for men), euphoric (*p* = 0.02; higher for men), and depressed (*p* = 0.0002; higher for men).

Time effects were noted for the following variables: attentive (*p* < 0.0001), tense (*p* = 0.006), bright (*p* = 0.005), alert (*p* < 0.0001), groggy (*p* < 0.0001), focused (*p* < 0.0001), energetic (*p* = 0.0001), and lethargic (*p* = 0.0002). For the above variables, values were generally different at Hours 2, 3, and 4 compared to pre meal. No other effects of statistical significance were noted for any variable (*p* > 0.05). Data are provided in Table 3. 

**Table 3:** Subjective feelings of 20 healthy men and women before and following ingestion of TheaTrim, placebo, or caffeine.

Variable	Pre Meal	Post Meal	Hour 1	Hour 2	Hour 3	Hour 4
Attentive men *^,†^						
TheaTrim	5.5 ± 0.5	5.8 ± 0.5	6.6 ± 0.5	6.8 ± 0.4	6.4 ± 0.5	6.5 ± 0.5
Placebo	5.5 ± 0.5	5.7 ± 0.4	6.3 ± 0.4	6.3 ± 0.4	6.1 ± 0.4	6.0 ± 0.4
Caffeine	5.2 ± 0.5	5.5 ± 0.5	5.9 ± 0.5	6.5 ± 0.4	6.5 ± 0.5	6.3 ± 0.6
Attentive women *^,†^						
TheaTrim	4.6 ± 0.6	5.5 ± 0.6	5.3 ± 0.5	6.2 ± 0.5	6.6 ± 0.5	6.9 ± 0.4
Placebo	4.2 ± 0.6	4.6 ± 0.7	5.0 ± 0.7	5.3 ± 0.6	5.8 ± 0.6	6.3 ± 0.6
Caffeine	4.8 ± 0.7	5.1 ± 0.5	4.7 ± 0.6	5.5 ± 0.6	5.7 ± 0.6	6.1 ± 0.8
Tense *men* ^†^						
TheaTrim	1.8 ± 0.4	1.4 ± 0.5	1.6 ± 0.6	1.2 ± 0.3	1.2 ± 0.3	1.1 ± 0.3
Placebo	2.3 ± 0.6	1.2 ± 0.3	1.2 ± 0.5	1.1 ± 0.4	0.8 ± 0.3	1.1 ± 0.4
Caffeine	2.2 ± 0.7	1.9 ± 0.7	1.2 ± 0.5	1.3 ± 0.5	1.4 ± 0.6	1.4 ± 0.6
Tense *women* ^†^						
TheaTrim	1.9 ± 0.6	2.1 ± 0.7	1.7 ± 0.5	1.2 ± 0.4	0.9 ± 0.3	1.0 ± 0.3
Placebo	1.2 ± 0.3	0.6 ± 0.2	0.8 ± 0.2	0.9 ± 0.3	0.6 ± 0.2	0.7 ± 0.3
Caffeine	1.9 ± 0.6	1.4 ± 0.5	0.7 ± 0.2	0.8 ± 0.3	0.8 ± 0.3	1.5 ± 0.7
Bright *men* ^†^						
TheaTrim	5.0 ± 0.5	4.5 ± 0.7	5.1 ± 0.8	5.6 ± 0.6	5.1 ± 0.6	5.3 ± 0.7
Placebo	5.2 ± 0.5	5.2 ± 0.6	5.8 ± 0.5	5.8 ± 0.6	5.5 ± 0.6	5.5 ± 0.6
Caffeine	4.2 ± 0.8	4.5 ± 0.6	4.9 ± 0.5	5.2 ± 0.6	5.4 ± 0.6	5.2 ± 0.6
Bright *women* ^†^						
TheaTrim	4.0 ± 0.8	4.4 ± 0.8	4.8 ± 0.6	5.4 ± 0.6	5.8 ± 0.7	5.8 ± 0.6
Placebo	3.4 ± 0.5	4.3 ± 0.6	4.0 ± 0.6	4.5 ± 0.6	5.1 ± 0.5	5.3 ± 0.6
Caffeine	4.0 ± 0.7	4.6 ± 0.5	4.3 ± 0.7	5.0 ± 0.7	5.4 ± 0.7	5.8 ± 0.8
Shaky *men*						
TheaTrim	1.0 ± 0.3	0.6 ± 0.2	1.4 ± 0.3	0.9 ± 0.3	0.9 ± 0.3	0.7 ± 0.2
Placebo	0.8 ± 0.4	0.8 ± 0.3	0.7 ± 0.3	0.6 ± 0.2	0.5 ± 0.2	0.5 ± 0.2
Caffeine	1.4 ± 0.5	1.0 ± 0.4	0.9 ± 0.5	0.9 ± 0.2	1.2 ± 0.6	0.8 ± 0.3
Shaky *women*						
TheaTrim	0.4 ± 0.1	0.5 ± 0.2	0.7 ± 0.2	1.3 ± 0.4	1.2 ± 0.4	0.9 ± 0.3
Placebo	1.1 ± 0.6	0.6 ± 0.2	0.6 ± 0.2	0.6 ± 0.2	0.7 ± 0.3	0.7 ± 0.3
Caffeine	0.6 ± 0.2	0.8 ± 0.3	0.7 ± 0.2	0.9 ± 0.3	1.3 ± 0.7	1.4 ± 0.7
Alert men *^,†^						
TheaTrim	5.9 ± 0.6	5.9 ± 0.5	6.5 ± 0.5	6.6 ± 0.4	6.2 ± 0.5	6.7 ± 0.5
Placebo	5.5 ± 0.5	5.7 ± 0.4	6.1 ± 0.5	6.2 ± 0.5	6.0 ± 0.4	5.8 ± 0.4
Caffeine	5.2 ± 0.5	4.9 ± 0.7	5.8 ± 0.4	6.3 ± 0.5	6.1 ± 0.4	5.8 ± 0.6
Alert women *^,†^						
TheaTrim	4.5 ± 0.6	4.6 ± 0.5	5.3 ± 0.6	5.9 ± 0.4	6.5 ± 0.4	6.7 ± 0.4
Placebo	4.0 ± 0.7	4.7 ± 0.7	4.8 ± 0.7	5.1 ± 0.7	5.9 ± 0.6	6.3 ± 0.6
Caffeine	4.5 ± 0.8	4.4 ± 0.5	4.7 ± 0.7	5.8 ± 0.7	5.7 ± 0.7	6.0 ± 0.8
Groggy *men* ^†,‡^						
TheaTrim	3.0 ± 0.6	2.3 ± 0.6	1.6 ± 0.5	1.6 ± 0.6	1.4 ± 0.4	1.3 ± 0.5
Placebo	3.5 ± 0.8	2.8 ± 0.8	1.7 ± 0.6	1.2 ± 0.3	2.0 ± 0.7	1.7 ± 0.5
Caffeine	4.3 ± 0.8	3.6 ± 0.9	2.4 ± 0.7	1.9 ± 0.6	1.7 ± 0.5	1.8 ± 0.6
Groggy *women* ^†,‡^						
TheaTrim	2.9 ± 0.6	3.0 ± 0.6	2.7 ± 0.6	1.5 ± 0.5	0.9 ± 0.3	0.9 ± 0.3
Placebo	3.5 ± 0.8	3.1 ± 0.7	2.8 ± 0.8	2.1 ± 0.5	1.9 ± 0.4	1.4 ± 0.4
Caffeine	3.8 ± 0.6	2.9 ± 0.5	2.7 ± 0.4	1.6 ± 0.4	1.9 ± 0.4	1.5 ± 0.5
Focused men *^,†^						
TheaTrim	5.7 ± 0.5	5.8 ± 0.5	6.4 ± 0.5	6.5 ± 0.5	6.4 ± 0.5	6.7 ± 0.5
Placebo	5.2 ± 0.5	5.5 ± 0.5	6.1 ± 0.4	6.0 ± 0.5	5.9 ± 0.5	5.7 ± 0.4
Caffeine	4.9 ± 0.5	5.4 ± 0.5	5.9 ± 0.4	6.4 ± 0.5	6.1 ± 0.4	6.0 ± 0.6
Focused women *^,†^						
TheaTrim	4.1 ± 0.5	4.8 ± 0.7	4.9 ± 0.6	5.5 ± 0.6	5.9 ± 0.5	6.5 ± 0.4
Placebo	3.8 ± 0.5	4.5 ± 0.6	4.7 ± 0.7	4.7 ± 0.7	5.6 ± 0.6	6.0 ± 0.4
Caffeine	4.3 ± 0.8	4.5 ± 0.5	4.5 ± 0.7	5.3 ± 0.6	5.6 ± 0.6	6.2 ± 0.8
Jittery *men* ^‡^						
TheaTrim	0.7 ± 0.2	0.7 ± 0.2	1.1 ± 0.3	0.8 ± 0.3	0.9 ± 0.3	0.8 ± 0.3
Placebo	0.8 ± 0.4	0.8 ± 0.3	0.7 ± 0.3	0.4 ± 0.2	0.5 ± 0.2	0.6 ± 0.2
Caffeine	1.3 ± 0.5	1.0 ± 0.4	1.0 ± 0.4	0.6 ± 0.2	1.5 ± 0.7	0.9 ± 0.5
Jittery *women* ^‡^						
TheaTrim	0.6 ± 0.2	0.6 ± 0.2	0.7 ± 0.2	1.7 ± 0.5	2.1 ± 0.7	1.2 ± 0.5
Placebo	1.0 ± 0.5	0.5 ± 0.2	0.6 ± 0.2	0.6 ± 0.2	0.7 ± 0.3	0.8 ± 0.3
Caffeine	1.3 ± 0.5	0.9 ± 0.3	1.0 ± 0.4	1.2 ± 0.6	1.4 ± 0.6	1.5 ± 0.7
Energetic men *^,†^						
TheaTrim	5.4 ± 0.5	5.0 ± 0.6	5.6 ± 0.7	5.7 ± 0.6	5.6 ± 0.5	5.5 ± 0.6
Placebo	4.3 ± 0.6	4.7 ± 0.7	5.3 ± 0.5	5.3 ± 0.6	5.3 ± 0.6	5.2 ± 0.5
Caffeine	4.3 ± 0.5	4.7 ± 0.4	5.2 ± 0.5	6.1 ± 0.4	5.7 ± 0.5	5.3 ± 0.7
Energetic women *^,†^						
TheaTrim	3.1 ± 0.6	4.1 ± 0.5	4.3 ± 0.6	5.1 ± 0.6	5.2 ± 0.8	6.0 ± 0.6
Placebo	3.1 ± 0.8	3.4 ± 0.9	3.3 ± 0.8	3.9 ± 0.5	4.7 ± 0.5	5.1 ± 0.7
Caffeine	3.4 ± 0.8	4.1 ± 0.6	4.0 ± 0.9	5.2 ± 0.9	5.1 ± 0.8	5.5 ± 0.8
Lethargic *men* ^†^						
TheaTrim	3.2 ± 0.7	2.7 ± 0.7	1.6 ± 0.5	1.9 ± 0.6	1.5 ± 0.5	1.6 ± 0.5
Placebo	3.5 ± 0.9	2.6 ± 0.8	1.7 ± 0.6	1.3 ± 0.4	2.1 ± 0.7	2.3 ± 0.6
Caffeine	4.0 ± 0.7	3.3 ± 0.8	2.7 ± 0.7	2.2 ± 0.6	1.8 ± 0.6	2.2 ± 0.7
Lethargic *women* ^†^						
TheaTrim	2.3 ± 0.5	2.5 ± 0.5	2.4 ± 0.6	1.6 ± 0.6	1.7 ± 0.6	1.3 ± 0.6
Placebo	3.1 ± 0.8	3.2 ± 0.5	2.3 ± 0.4	2.0 ± 0.6	1.8 ± 0.5	1.9 ± 0.6
Caffeine	3.2 ± 0.7	2.3 ± 0.6	2.4 ± 0.5	2.4 ± 0.7	2.1 ± 0.7	2.4 ± 0.7
Euphoric men *						
TheaTrim	3.4 ± 0.9	3.3 ± 1.0	3.5 ± 0.9	3.7 ± 0.9	3.7 ± 0.9	3.6 ± 1.0
Placebo	3.9 ± 0.7	3.4 ± 0.8	3.4 ± 0.8	3.3 ± 1.0	3.4 ± 0.8	3.5 ± 0.8
Caffeine	3.2 ± 0.7	3.3 ± 0.9	3.4 ± 1.0	3.5 ± 0.9	3.5 ± 0.9	3.3 ± 0.9
Euphoric women *						
TheaTrim	2.4 ± 0.7	2.6 ± 0.8	2.5 ± 0.8	2.9 ± 0.9	2.7 ± 0.9	2.9 ± 1.0
Placebo	2.5 ± 0.7	2.7 ± 0.9	2.6 ± 0.8	2.6 ± 0.8	2.6 ± 0.9	2.8 ± 1.0
Caffeine	2.7 ± 0.8	2.8 ± 0.8	2.8 ± 0.9	3.0 ± 0.9	3.6 ± 1.0	3.5 ± 1.0
Depressed men *^,‡^						
TheaTrim	0.8 ± 0.2	0.8 ± 0.2	0.8 ± 0.2	0.8 ± 0.2	0.7 ± 0.2	0.7 ± 0.2
Placebo	0.7 ± 0.2	0.6 ± 0.2	0.4 ± 0.1	0.4 ± 0.1	0.4 ± 0.1	0.5 ± 0.1
Caffeine	0.8 ± 0.2	0.7 ± 0.2	0.7 ± 0.2	0.9 ± 0.5	0.8 ± 0.2	0.6 ± 0.2
Depressed women *^,‡^						
TheaTrim	0.5 ± 0.2	0.3 ± 0.1	0.3 ± 0.1	0.3 ± 0.1	0.3 ± 0.1	0.3 ± 0.1
Placebo	0.5 ± 0.2	0.3 ± 0.1	0.4 ± 0.2	0.5 ± 0.2	0.4 ± 0.2	0.4 ± 0.2
Caffeine	0.5 ± 0.2	0.6 ± 0.2	0.5 ± 0.2	0.5 ± 0.2	0.5 ± 0.2	1.0 ± 0.5

Data are presented separately for men and women to best inform readers about how each sex responded. Values are mean ± SEM (standard error of the mean). * Sex effect for attentiveness (*p* = 0.0003); men higher than women; * Sex effect for alert (*p* = 0.0005); men higher than women; * Sex effect for focused (*p* < 0.0001); men higher than women; * Sex effect for energetic (*p* < 0.0001); men higher than women; * Sex effect for euphoric (*p* = 0.02); men higher than women; * Sex effect for depressed (*p* = 0.0002); men higher than women; ^†^ Time effect for attentive (*p* < 0.0001); ^†^ Time effect for tense (*p* = 0.006); ^†^ Time effect for bright (*p* = 0.005); ^†^ Time effect for alert (*p* < 0.0001); ^†^ Time effect for groggy (*p* < 0.0001); ^†^ Time effect for focused (*p* < 0.0001); ^†^ Time effect for energetic (*p* = 0.0001); ^†^ Time effect for lethargic (*p* = 0.0002); ^‡^ Condition effect for groggy (*p* = 0.04); ^‡^ Condition effect for jittery (*p* = 0.01); ^‡^ Condition effect for depressed (*p* = 0.02). No other differences of statistical significance were noted for any variable (*p* > 0.05).

### 3.3. Cognitive Performance and Reaction Time

Regarding the DSST, a time effect was noted (*p* = 0.03), with values higher at Hour 4 compared to post meal. Regarding the TMT, a time effect was noted (*p* = 0.02), with values higher at pre meal compared to Hour 2. No other effects of statistical significance were noted for DSST or TMT (*p* > 0.05). For reaction time, no effects of statistical significance were noted (*p* > 0.05). Data are provided in Table 4. 

**Table 4:** Trail Making Test (TMT), Digit Symbol Substitution Test (DSST), and reaction time scores of 20 healthy men and women before and following ingestion of TheaTrim, placebo, or caffeine.

Variable	Pre Meal	Post Meal	Hour 1	Hour 2	Hour 3	Hour 4
TMT (s) *men *^†^						
TheaTrim	23.0 ± 1.7	25.3 ± 3.3	19.9 ± 1.4	21.6 ± 2.7	19.9 ± 1.8	22.5 ± 2.7
Placebo	25.3 ± 2.7	25.0 ± 3.0	23.6 ± 3.6	21.8 ± 1.9	22.2 ± 2.3	23.5 ± 2.0
Caffeine	28.2 ± 7.5	25.4 ± 2.0	21.1 ± 1.4	21.5 ± 2.6	22.0 ± 2.4	24.5 ± 2.8
TMT (s) *women* ^†^						
TheaTrim	32.4 ± 5.2	25.0 ± 2.5	23.3 ± 1.8	20.9 ± 2.1	25.0 ± 3.6	24.7 ± 2.0
Placebo	25.0 ± 2.6	23.5 ± 2.8	21.9 ± 2.6	21.0 ± 2.3	26.3 ± 2.8	28.0 ± 4.4
Caffeine	25.0 ± 2.3	26.8 ± 2.9	22.4 ± 2.0	21.1 ± 1.9	26.4 ± 4.1	25.1 ± 2.3
DSST *men *^†^						
TheaTrim	96.0 ± 2.6	95.8 ± 3.5	99.4 ± 3.7	101.7 ± 4.2	102.2 ± 3.2	104.5 ± 3.3
Placebo	97.7 ± 4.9	100.0 ± 3.4	99.1 ± 3.5	101.3 ± 5.0	102.8 ± 3.2	101.6 ± 3.9
Caffeine	96.1 ± 4.9	95.4 ± 4.0	101.1 ± 3.0	101.1 ± 4.7	103.2 ± 3.5	104.7 ± 4.5
DSST *women *^†^						
TheaTrim	104.3 ± 5.3	93.9 ± 3.2	98.1 ± 4.0	102.6 ± 5.1	103.3 ± 4.1	104.5 ± 4.6
Placebo	109.1 ± 5.0	98.7 ± 2.3	97.9 ± 3.4	102.1 ± 3.5	97.5 ± 3.0	104.7 ± 3.8
Caffeine	103.7 ± 5.1	96.2 ± 3.9	98.9 ± 2.9	98.3 ± 3.5	100.0 ± 4.8	106.6 ± 3.9
Reaction Time (s) *men*						
TheaTrim	0.419 ± 0.02	0.414 ± 0.01	0.408 ± 0.02	0.389 ± 0.01	0.395 ± 0.02	0.400 ± 0.01
Placebo	0.441 ± 0.02	0.433 ± 0.02	0.414 ± 0.02	0.415 ± 0.02	0.424 ± 0.02	0.410 ± 0.02
Caffeine	0.437 ± 0.02	0.417 ± 0.02	0.403 ± 0.02	0.404 ± 0.01	0.401 ± 0.01	0.415 ± 0.02
Reaction Time (s) *women*						
TheaTrim	0.427 ± 0.02	0.434 ± 0.02	0.414 ± 0.02	0.419 ± 0.02	0.409 ± 0.02	0.404 ± 0.02
Placebo	0.416 ± 0.01	0.416 ± 0.02	0.408 ± 0.02	0.436 ± 0.02	0.398 ± 0.02	0.439 ± 0.02
Caffeine	0.436 ± 0.03	0.427 ± 0.02	0.431 ± 0.03	0.402 ± 0.02	0.427 ± 0.02	0.419 ± 0.02

Data are presented separately for men and women to best inform readers about how each sex responded. Values are mean ± SEM (standard error of the mean). ^†^ Time effect for DSST (*p* = 0.03); Hour 4 higher than pre meal; ^†^ Time effect for TMT (*p* = 0.03); pre meal higher than Hour 2; No other differences of statistical significance were noted (*p* > 0.05).

### 3.4. Heart Rate, Blood Pressure, and Rate Pressure Product

With regards to heart rate, the following was noted: sex effect (*p* < 0.0001; women higher than men), time effect (*p* = 0.0003; Hours 2, 3, and 4 are lower than all other times). For systolic blood pressure, the following was noted: sex effect (*p* < 0.0001; men higher than women), condition effect (*p* = 0.0008; TheaTrim higher than placebo). No time effect was noted, with little change from pre to post meal noted for any condition. For diastolic blood pressure, no effects of statistical significance were noted (*p* > 0.05). For rate pressure product, the following was noted: sex effect (*p* = 0.0004; women higher than men), time effect (*p* = 0.0005; Hours 2, 3, and 4 are lower than all other times). No other effects of statistical significance were noted for any variable (*p* > 0.05). Data are provided in Table 5.

**Table 5:** Heart rate, blood pressure, and rate pressure product of 20 healthy men and women before and following ingestion of TheaTrim, placebo, or caffeine.

Variable	Pre Meal	Post Meal	Hour 1	Hour 2	Hour 3	Hour 4
Heart Rate (bpm) men *^,†^						
TheaTrim	66.7 ± 3.4	65.4 ± 3.6	65.1 ± 3.1	60.6 ± 3.1	59.1 ± 3.0	60.0 ± 3.1
Placebo	67.1 ± 3.0	67.3 ± 3.2	64.8 ± 3.5	60.1 ± 4.0	58.6 ± 3.1	57.0 ± 2.8
Caffeine	66.4 ± 3.4	63.8 ± 2.9	61.2 ± 2.6	57.0 ± 2.5	55.8 ± 2.6	57.3 ± 3.5
Heart Rate (bpm) women *^,†^						
TheaTrim	73.8 ± 3.3	73.5 ± 3.7	68.6 ± 3.6	70.7 ± 3.8	68.2 ± 4.4	67.7 ± 4.5
Placebo	77.5 ± 3.6	75.3 ± 3.4	73.4 ± 3.8	70.8 ± 4.2	70.7 ± 5.0	73.4 ± 5.0
Caffeine	77.9 ± 4.1	73.7 ± 3.5	69.5 ± 4.3	68.7 ± 4.3	68.7 ± 5.6	68.6 ± 4.5
SBP (mm Hg) men *^,‡^						
TheaTrim	129.2 ± 3.2	124.6 ± 2.7	126.7 ± 4.2	128.3 ± 3.4	129.4 ± 3.8	125.4 ± 3.6
Placebo	126.3 ± 3.1	125.0 ± 3.0	123.0 ± 3.3	120.4 ± 4.1	120.6 ± 2.7	123.0 ± 3.1
Caffeine	126.6 ± 1.9	129.0 ± 2.4	127.6 ± 1.6	125.0 ± 3.0	127.7 ± 2.6	128.5 ± 1.9
SBP (mm Hg) women *^,‡^						
TheaTrim	119.5 ± 2.7	118.1 ± 2.5	121.6 ± 3.0	121.7 ± 3.3	119.3 ± 3.8	120.2 ± 3.0
Placebo	116.1 ± 3.1	114.5 ± 2.4	115.5 ± 2.4	114.0 ± 3.9	114.8 ± 3.9	111.7 ± 3.8
Caffeine	115.0 ± 2.1	112.1 ± 3.2	114.1 ± 2.0	114.9 ± 4.0	114.6 ± 3.3	117.2 ± 4.7
DBP (mm Hg) *men*						
TheaTrim	72.0 ± 3.3	64.8 ± 3.7	70.8 ± 3.5	70.6 ± 2.6	68.9 ± 3.2	71.3 ± 3.3
Placebo	69.1 ± 2.3	68.0 ± 2.6	69.2 ± 3.7	66.9 ± 3.8	67.9 ± 2.9	68.5 ± 3.3
Caffeine	72.1 ± 1.5	70.7 ± 1.8	70.5 ± 2.7	72.1 ± 1.9	74.0 ± 2.8	70.8 ± 2.5
DBP (mm Hg) *women*						
TheaTrim	69.9 ± 1.9	69.4 ± 2.4	69.0 ± 2.6	70.6 ± 2.4	71.4 ± 2.3	70.1 ± 2.3
Placebo	66.7 ± 2.1	68.1 ± 1.9	65.9 ± 2.2	67.9 ± 3.0	68.4 ± 3.5	67.3 ± 2.6
Caffeine	69.5 ± 2.0	65.2 ± 1.9	67.3 ± 2.4	71.3 ± 2.8	68.9 ± 2.7	67.4 ± 2.4
RPP men *^,†^						
TheaTrim	8636.4 ± 520	8151.8 ± 506	8215.4 ± 416	7743.1 ± 395	7633.0 ± 438	7467.3 ± 308
Placebo	8493.5 ± 481	8379.4 ± 390	7950.0 ± 447	7224.4 ± 524	7064.9 ± 401	6979.0 ± 325
Caffeine	8395.1 ± 426	8217.0 ± 388	7814.8 ± 366	7091.9 ± 273	7111.0 ± 324	7318.1 ± 386
RPP women *^,†^						
TheaTrim	8830.1 ± 445	8669.7 ± 449	8319.8 ± 436	8614.3 ± 529	8127.0 ± 559	8172.0 ± 624
Placebo	9012.4 ± 499	8638.6 ± 458	8496.7 ± 503	8088.8 ± 572	8162.7 ± 685	8262.7 ± 713
Caffeine	8959.2 ± 502	8243.1 ± 422	7942.7 ± 540	7912.2 ± 576	7887.0 ± 695	8040.2 ± 620

Data are presented separately for men and women to best inform readers about how each sex responded. Values are mean ± SEM. SBP = systolic blood pressure; DBP = diastolic blood pressure; RPP = rate pressure product. * Sex effect for heart rate (*p* < 0.0001); women higher than men; * Sex effect for SBP (*p* < 0.0001); men higher than women; * Sex effect for RPP (*p* = 0.0004); women higher than men; ^†^ Time effect for heart rate (*p* = 0.0003); Hours 2, 3, and 4 are lower than all other times; ^†^ Time effect for RPP (*p* = 0.0005); Hours 2, 3, and 4 are lower than all other times; ^‡^ Condition effect for SBP (*p* = 0.0008; TheaTrim higher than placebo). No other differences of statistical significance were noted for any variable (*p* > 0.05).

## 4. Discussion

Data from the present investigation indicate that within a controlled laboratory setting: (1) TheaTrim or caffeine do not improve cognitive performance or reaction time in a statistically significant manner; (2) TheaTrim improves a variety of subjective feelings related to energy and mood; and (3) TheaTrim or caffeine do not elevate heart rate or blood pressure in a statistically significant manner. These findings are in reference to a sample of healthy, young men and women who regularly consume caffeinated products.

Contrary to our hypotheses, neither caffeine nor TheaTrim produced significant effects on the neurocognitive tests. There are a variety of reasons this could be the case but we believe that the time of day of testing could be the variable that contributed most to our findings. That is, subjects appeared to be more alert and focused as the testing day progressed, regardless of condition. This likely impacted our findings, as attention is known to influence task engagement and performance [24,25]. In short, it may have been difficult for some of our subjects to perform well in the cognitive tests when being asked to do so at such an early time of day (e.g., 6:00 or 7:00 a.m.). Further research will need to explore the possible impact of TheaTrim on cognitive performance assessed at differing times of day (e.g., afternoon or evening). Moreover, while our cognitive test battery included three well-utilized tasks (*i.e.*, TMT, DSSST, and computerized reaction time), alternatives to these tests may be considered in future evaluations of theacrine-containing dietary supplements. Aside from cognitive performance, it is possible that the cardiovascular variables may have been differently impacted based on the time of day that measurements were obtained [26]. Future studies investigating the impact of dietary supplements to impact cognitive and cardiovascular variables may consider the influence of time of day on the outcomes. 

Aside from the timing of treatment possibly impacting our findings, it is possible that the dosage of theacrine and/or caffeine could have been too low. That being said, prior work with theacrine at a similar dosage as used in the present study yielded positive findings in overall mood. Of course, the relatively small sample size and variance between participants could have impacted our ability to detect changes of statistical significance. These issues should be considered along with other limitations, as mentioned later in this section. 

Our data for subjective feelings (Table 3) support the existing research involving theacrine use in human subjects [8]. The current study indicates trends for higher self-report measures of attentiveness, alertness, focus, and energy, with lower scores noted for lethargic in the TheaTrim condition compared to the caffeine and placebo conditions. TheaTrim also showed a significantly lower measure for “groggy” compared to the caffeine and placebo conditions. Time effects were noted for the following variables: attentive tense, bright, alert, groggy, focused, energetic, and lethargic. These findings suggest that subjects generally felt better as the testing protocol progressed through the morning hours.

Although subjective, the findings of increased attentiveness, alertness, focus, and energy should not be disregarded, as such subjective feelings have been demonstrated to be associated to a variety of positive outcomes. It has been reported that positive affect can favorably influence complex decision making/ability to solve problems [27], which may have implications in a wide variety of situations. Specifically, improving subjective measures of overall mood may positively impact academic performance [28], workplace performance [29], and athletic performance [30]. 

Some earlier studies focused on caffeine consumption were conducted to determine the possible cardiovascular consequences of routine caffeine intake. For example, Rosenberg *et al.* reported that caffeine consumption (via daily cups of coffee) increased the risk of myocardial infarction in men under the age of 55 [31]. However, most other studies indicate that caffeine poses little or no risk to the development of cardiovascular disease, particularly coronary heart disease [32,33,34]. 

Clearly, caffeine is widely used worldwide by children, adolescents, and adults [35,36], with little to no cardiovascular consequence, in particular when used at dosages less than 400 mg/day [37]. Our data using caffeine in isolation and in conjunction with other ingredients support the overall safety of caffeine, evidenced by no change in heart rate or blood pressure with acute ingestion. In fact, no differences were noted in heart rate response for either men or women when ingesting caffeine, TheaTrim, or placebo. Systolic and diastolic blood pressure exhibited negligible changes during the post ingestion period (Table 5). Perhaps most importantly, the rate pressure product was lower following condition administrations as compared to prior to administration, suggesting decreased myocardial work. 

While our sample size was relatively small and no significant interaction effects were noted for the above tests, there were some minor improvements with the TheaTrim condition that should be noted. For example, male subjects using TheaTrim improved performance on the TMT by roughly 17% compared to an improvement of 12% under the caffeine condition (comparing post meal time to completion to the average of the times for each of the 4-h testing periods). Similarly, men showed an improvement in reaction time by 6% using the TheaTrim condition compared to 4% under the caffeine condition (comparing post meal reaction time to the best, or lowest time of the following 4 h; for TheaTrim, this occurred at Hour 2, while caffeine occurred at Hour 3). In women, comparing post meal reaction time to the average reaction time over the 4-h testing period, the TheaTrim condition showed a performance improvement of 5% while caffeine showed an improvement of 2%. While these changes and differences may be small, they provide some evidence for a possible effect of TheaTrim on the noted variables in both men and women, who are noted to have similar and differential responses in cognitive performance tasks and processing, depending on outcome measure [38,39]. Future studies inclusive of a larger sample size are needed to more fully elucidate the impact of this supplement on cognitive performance and reaction time in men and women. 

Although dietary data were different between men and women, values were relatively stable across conditions, indicating that dietary intake was unlikely to impact our findings. This is supported by the fact that all subjects reported to all testing sessions in a fasted state and received a standardized meal. Appetite rose throughout the testing session, as was expected, given the prior fasting and the relatively small meal administered to the subjects. While it has been noted that caffeine ingestion at 300 mg may limit food intake in men (but not in women) [40], the present study using a relatively low dosage of caffeine did not find any anorectic effects of caffeine (or TheaTrim) in either men or women. This may be due to the fact that subjects were relatively hungry upon arrival to the lab and only received a small liquid meal during their visit, which was likely inadequate to satisfy their appetite, regardless of which condition they received. 

Some limitations of this work should be noted. First and as discussed above, subjects reported to the lab in the early morning hours and this may have negatively impacted their cognitive performance and mood. Although the time of day for testing was matched for all visits, participation during the late morning or early afternoon hours may have resulted in different findings. Second and mentioned above, the current study examined a relatively small sample of only 10 men and 10 women. Considering the potential confounding variance in circadian typology [41,42], the small sample could have masked our ability to detect statistically significant findings. Third, although we believe our inclusion of outcome measures was appropriate, it was in no way comprehensive in terms of assessment of cognitive ability. Additional tests of cognitive performance could have been included and may have been noted to differ depending on the assigned treatment. Finally, because the tested supplement contained a variety of ingredients (as indicated in the Methods section), it is unknown what independent contribution theacrine may have had on the chosen outcome measures. Future work is needed using theacrine alone, perhaps compared to other known mood-enhancing agents, to determine the impact of theacrine on mood and related variables. The above limitations should be considered when designing future experiments within this area of investigation. 

While the validity of the DSST and TMT are well-established, other cognitive testing procedures may need to be examined. As mentioned in the introduction, one study noted significant improvements in the RVIP task under the influence of caffeine [12]. As mentioned above, time effects have been noted for cardiovascular variables and cognitive performance without supplementation, so this may also be a confounding factor. Aden and colleagues have suggested that controlling for circadian typology may be useful in eliminating this confounding factor [42]. Examining a range of dosages for theacrine in a larger cohort may also be warranted. As discussed in the introduction, 200 mg was shown to be more effective than 100 mg and 400 mg in a smaller sample of human subjects [8]. 

## 5. Conclusions

In conclusion, the current study was, to our knowledge, only the third human trial to evaluate the ingredient known as theacrine—done within a multi-ingredient dietary supplement. Our data indicate that acute intake of the theacrine-containing dietary supplement TheaTrim does not significantly alter heart rate or blood pressure in healthy men or women. Moreover, neither TheaTrim nor caffeine alone improves cognitive performance in a statistically significant manner. However, TheaTrim does appear to favorably impact multiple subjective feelings related to energy and mood compared to caffeine and a placebo. Future work is needed to extend these initial findings, perhaps using theacrine alone as well as within multi-ingredient dietary supplements, in an attempt to expand our understanding of theacrine use by human subjects.
